# COVID-19: The Hidden Impact on Mental Health and Drug Addiction

**DOI:** 10.3389/fpsyt.2020.00767

**Published:** 2020-07-29

**Authors:** Stefania Chiappini, Amira Guirguis, Ann John, John Martin Corkery, Fabrizio Schifano

**Affiliations:** ^1^ Psychopharmacology, Drug Misuse & Novel Psychoactive Substances Research Unit, School of Life and Medical Sciences, University of Hertfordshire, Hatfield, United Kingdom; ^2^ Swansea University Medical School, Swansea University, Swansea, United Kingdom

**Keywords:** COVID-19, addiction, mental health, drug abuse, prescription drug misuse

## Introduction

There is concern the Coronavirus Disease (COVID)-19 pandemic is having a negative impact on the mental health of the general population through a range of suggested mechanisms: fear, uncertainty, and anxiety; social distancing/isolation; loneliness; and economic repercussions (1–3). Previous disasters such as the Severe Acute Respiratory Syndrome (SARS) in 2003 (4–6) contributed to increased anxiety, mood, and thought disorders, adjustment disorders, and post-traumatic stress disorders (PTSD) (1, 7–15), resulting, in extreme cases, in suicidal behaviours (e.g., suicidal ideation, suicide attempts, and actual suicide) (10, 16), especially in cases of concomitant Substance Use Disorder (SUD) (17, 18). According to a recent study from the Well Being Trust (18) the high levels of stress, isolation and unemployment due to the COVID-19 pandemic could cause up to 75,000 “deaths of despair” related to deaths to drug, alcohol, and suicide (18). High risk of mental illness was previously identified in individuals with existing or history of mental illnesses (1, 9, 12, 14, 19), but also vulnerable categories might be considered the elderly (>80 years old), children/adolescents, individuals from deprived areas, peri-natal women and BAME (Black, Asian and minority ethnicities) (1, 12, 14, 19). Finally, healthcare workers have been experiencing emotional overload due to several reasons, including both organizational issues relating to the shortage of suitable personal protective equipment, reduction in human resources and relentless work shifts (20–23), but also the burden developed by the fear of becoming infected and infecting relatives, high mortality rates, grieving the loss of patients and colleagues, separation from families (22–24). Specifically, according to Huang et al. (25), among the first-line medical staff of a Tertiary Infectious Disease Hospital for COVID-19 in China, the incidence of anxiety and post traumatic symptoms in female medical staff was higher than that in male, and in nurses more represented than that in doctors (25).

## Discussion

Often overlooked in this scenario are those with SUD (26, 27), who may experience: (a) changes in levels of drug use—an increase is often seen as a reactive behaviour to negative impact of disasters; (b) a shift to other substances if access to those previously used become limited; (c) a relapse, if they had already recovered from alcohol/drug addiction. Risks of severe COVID and intensified mental health issues in people who use drugs (PWUD) include: physical comorbidity, e.g., lung or cardiovascular disease, HIV, viral hepatitis infections; psychological comorbidity, e.g., general distress, sleep disorders, anxiety/mood disorders, psychotic symptoms; and homelessness, incarceration, economic difficulties, and socioeconomic issues deriving from drug addiction (8, 11, 27, 28). Overdose risk for addicted people who are home-isolating, and hence with typically no one to inject them with naloxone, should be considered in a time of overloaded emergency services and healthcare systems in general (27, 29). The COVID-19 pandemic is already impacting drug markets, including shortages of numerous types of drugs at the street level, price increases for consumers on the black market and reductions in purity. Synthetic drugs’ availability, such as methamphetamine, is drastically reduced due to air travel restrictions and flight cancellations, while cocaine, mostly trafficked by sea, continues to be detected in European ports during the pandemic (30). Heroin and opioids seem to be pushed toward being trafficked along maritime routes. Finally, cannabis appears to be less available, due to restrictions on movement across regions and borders under coronavirus lockdown. These disruptions are likely to grow and further increase risks for people who use drugs, for example by increasing variability in drug purity, the likelihood of adulteration, and contamination of heroin supply with synthetic opioids, such as fentanyl. These issues can also encourage shifts to more at-risk drug using behaviours such as use of drugs such as street benzodiazepines, and synthetic cannabinoids (31). Additionally, the COVID-19 crisis is likely to increase the need to access drug treatment and services, e.g., extra demand for opioid substitution therapy and other medication. Access to drug services is being disrupted by self-quarantine, social distancing and other public health measures adopted for dealing with COVID-19 (27, 29, 31). Similarly, community pharmacies are challenged by staff shortages, service disorganisation, and self-isolation (27, 29, 32).

In response to the long-lasting and wide-ranging challenging effects of the pandemic (5, 12, 19, 27, 29), some harm-avoiding interventions have been adopted, including: more flexible take-home-medication treatment programmes for opioid addicted patients (33, 34); guidance for facilitating controlled substance prescribing (26, 29, 35); tele-health for monitoring drug-dependent patients; and access to virtual support groups through online meetings (15, 26, 32). Conversely, both peer-support groups and rehabilitation facilities have suspended programmes and limited new admissions (27, 32). Hollander & Carr (36) compared and contrasted the acceptability and impact of telemedicine versus in-person consultations. During the COVID pandemic, telehealth has demonstrated to enable continuity of services, while protecting service providers from infection. However, in-person consultations are still needed for certain groups of patients where maintenance in treatment is at risk.

In this context, due to the disruption of drug markets, reduced supply and access to illicit drugs, internet drug-seeking activities may be on the increase. In line with this, rogue/illicit pharmaceutical products, such as benzodiazepines, has also reportedly doubled their prices in some areas (24). Alternative drugs or medications might be considered by users including quetiapine, gabapentinoids, Z-drugs (e.g., zolpidem) (37–39) and some Over-The-Counter (OTC) medications (37, 38), such as codeine; ephedrine and pseudoephedrine; and the antidiarrhoeal loperamide (“poor man’s methadone”).

## Implications in Practice

Interventions addressing the health, psychological, and social effects of the pandemic are required. Healthcare professionals have an important role in educating patients about the common psychological effects of a pandemic. COVID-19, together with general environmental factors, such as stress or trauma, may contribute to both a mental illness and a SUD developing. A proactive approach to upscale our mental health care, emergency preparedness and response for people with SUDs is urgently needed; mental health services should develop and evaluate: clear remote assessment; care pathways for people at risk; psycho-education strategies, regarding self-harm/suicide, overdoses, and domestic violence; and staff training to support new ways of working (1, 7, 12). Healthcare providers, including pharmacists, and public health policies are challenged to: develop strategies to implement prevention measures against transmission of COVID-19 in drug users settings, such as preventing overcrowding or sharing drug-using equipment; and ensure continuity of care for drug-users and people with SUDs. Specifically, access to community maintenance, e.g., expand methadone delivery *via* mobile teams for quarantined patients should be facilitated (40, 41). Monitoring psychosocial needs and delivering psychosocial support to vulnerable patients as well as healthcare workers should be provided (2, 3, 8, 42, 43). It is crucial to strengthen telemedicine and support it with appropriate governance and funding in order to be able to monitor the mental health situation post-pandemic. Supporting healthcare workers with appropriate equipment, training on telehealth and caring for their safety with respect to protection against infection and spread of infection, preventing violence and burglary in drug treatment services, pharmacies would enable robust support against a possible mental health wave post-pandemic. Prescribers and pharmacists should be warned about: possible requests to prescribe more drugs than needed to take home; excessive sales of prescription/OTC products which might be diverted and abused; and aggression toward staff. Developing multidisciplinary support platforms could be helpful in reducing the mental distress due to misinformation and teaching problem-solving strategies to cope with the pandemic (13).

## Author Contributions

The opinion was developed by all authors. SC drafted the first version of the manuscript with input from all authors. All authors contributed to the article and approved the submitted version.

## Conflict of Interest

The authors declare that the research was conducted in the absence of any commercial or financial relationships that could be construed as a potential conflict of interest.
